# What Do We Know About Hypertrophic Cardiomyopathy in Poland in the Period 2016–2021 Based on Data From the Public Health System?

**DOI:** 10.3389/ijph.2026.1608693

**Published:** 2026-03-11

**Authors:** Małgorzata Niemiec, Maria Stec, Jacek Grzybowski, Urszula Cegłowska, Anna Wiśniewska, Kacper Hałgas, Kinga Czepczor, Maciej Podolski, Bartosz Gruchlik, Gabriela Orzeł-Łomozik, Daniel Cieśla, Mariusz Gąsior, Tomasz Hryniewiecki, Przemysław Leszek, Alida L. P. Caforio, Katarzyna Mizia-Stec

**Affiliations:** 1 Department of Cardiology, First Department of Cardiology, Medical University of Silesia in Katowice, Katowice, Poland; 2 European Reference Network on Rare Heart Diseases (ERN GUARD-HEART), Brussels, Belgium; 3 Department of Cardiomyopathies, National Institute of Cardiology, Warsaw, Poland; 4 Department of Analysis and Strategy, Ministry of Health of the Republic of Poland, Warsaw, Poland; 5 Department of Epidemiology and Health Promotion, Faculty of Public Health, Center for Postgraduate Education, Warsaw, Poland; 6 3rd Department and Clinic of Cardiology, Faculty of Medicine with the Division of Dentistry in Zabrze, Silesian Center for Heart Diseases, Medical University of Silesia, Katowice, Poland; 7 Department of Valvular Heart Disease, National Institute of Cardiology, Warsaw, Poland; 8 Department of Heart Failure and Transplantology, Laboratory for Mechanical Circulatory Support and Heart Transplantation, National Institute of Cardiology, Warsaw, Poland; 9 Cardiology, Department of Cardiac Thoracic Vascular Sciences and Public Health, University of Padova, Padova, Italy; 10 European Reference Network for Rare, Low Prevalence and Complex Diseases of the Heart-ERN GUARD-Heart, Brussels, Belgium

**Keywords:** cardiomyopathy, heart failure, hypertrophic cardiomyopathy, national health Fund, public health systems

## Abstract

**Objectives:**

The aim of the study was to comprehensively characterize the population of hypertrophic cardiomyopathy (HCM) patients in Poland in 2016–2021, including the analysis of annual incidence, prevalence, comorbidities, and therapeutic paths.

**Methods:**

A population-based cross-sectional study was conducted using data from the Polish National Health Fund database. The analysis included a registered annual incidence and prevalence rates, mortality, prognosis, and data on patients pathways in the healthcare system.

**Results:**

In 2016, the registered annual incidence of HCM diagnosis in Poland was 1,494 cases (3.89/100,000), decreasing to 808 cases (2.11/100,000) in 2021. Despite the decline in new diagnoses, the registered prevalence of HCM increased from 13,271 cases (34.53/100,000) in 2016 to 13,880 cases (36.23/100,000) in 2021, indicating improved patient survival. Annual mortality rose from 824 deaths (2.14/100,000) in 2016 to 1,022 deaths (2.67/100,000) in 2021, with the highest mortality observed in older adults (60+ years).

**Conclusion:**

The study highlights significant underdiagnosis of HCM in Poland. Mortality in HCM population remains high, underscoring the need for improvement in earlier detection and comprehensive care strategies in Polish healthcare system.

## Introduction

Hypertrophic cardiomyopathy (HCM) is a genetically diverse heart disease characterized by cardiac hypertrophy resulting from mutations in genes encoding sarcomere proteins. HCM is a significant cause of morbidity and mortality, leading to heart failure, cardiac arrhythmias and sudden cardiac death, especially among young people [1, 2].

Electrocardiography has historically played a fundamental role in the screening and early suspicion of hypertrophic cardiomyopathy, particularly in young and athletic populations. Landmark studies and European Society of Cardiology recommendations have demonstrated that electrocardiography is a sensitive and cost-effective first-line screening tool for identifying individuals who require further diagnostic evaluation [3–6]. Echocardiography remains the standard imaging modality for confirming the diagnosis, while cardiac magnetic resonance imaging and genetic testing provide complementary information in selected or diagnostically challenging cases [3–6].

Over the past 30 years, the number of disability-adjusted life years (DALYs) lost due to cardiomyopathy and myocarditis increased from 7.06 million (95% CI: 6.30–8.63) to 9.14 million (95% CI: 7.86–10.0). The global incidence of HCM is estimated at 1:250–500, which means that in Poland this disease may affect from 75,000 to 150,000 people [7]. Despite advances in diagnostics and screening technologies, the true burden of HCM is difficult to estimate due to underdiagnosis, misclassification, and differences in access to healthcare in different regions [7, 8]. This results in significant differences between theoretical estimates of incidence and actual data on the prevalence of HCM [9].

This problem is particularly visible where access to advanced diagnostic methods, such as cardiac magnetic resonance (CMR) or genetic testing, is limited. A better understanding of the epidemiology of HCM is crucial to improving treatment outcomes, as early diagnosis and risk assessment are the basis for effective disease management and reducing its complications.

The aim of this study is a comprehensive analysis of HCM in Poland, including the assessment of the registered annual incidence, prevalence and treatment outcomes of patients with a confirmed clinical diagnosis in 2016–2021, a period covering mainly the years before the outbreak of the COVID-19 pandemic. Using National Health Fund (NFZ) data and real-world clinical evidence, the study aims to highlight the current state of HCM diagnosis and therapy, identify existing disparities, and propose practical solutions to improve the quality of healthcare. Previous studies based on the public healthcare data have already been conducted, focusing on the general concept of cardiomyopathies [10] and patients pathways of diagnosis and treatment [11].

## Methods

This population-based, cross-sectional study utilized data from the Polish National Health Fund database, which comprehensively records healthcare data for the insured population in Poland. Patient records were identified using the International Classification of Diseases, 10th Revision (ICD-10) codes specific to hypertrophic cardiomyopathy, namely, I42.1 (obstructive hypertrophic cardiomyopathy) and I42.2 (non-obstructive hypertrophic cardiomyopathy) [8], covering the period from January 1, 2016, to December 31, 2021. Patients were included in the study if these codes appeared at any point during their medical history within the specified timeframe. Exclusion criteria involved ICD codes consistent with ischemic heart disease: I24, I25, I21, I20 at the time of CMs diagnosis. It was possible to match the information of individual patients through the hospital registry number and the national identification number.

The analysis focused on key parameters, including the first recorded diagnosis of HCM, and based on these data the registered incidence and prevalence. The presence of deaths was estimated based on data from the public healthcare system and the Ministry of Digitization from 2016 to 2021. The collected data also included information on the following services: UH - urgent hospitalisation due to exacerbation of the disease, EH - elective hospitalisation, TMC - tertiary out-patient medical care, GP - first-line out-patient healthcare. If the patient was hospitalized again due to cardiovascular diseases within 24 h, both hospitalizations were considered one admission [10, 11].

The data of registered comorbidities were also obtained.

The reliance on ICD-10 coding within the NFZ database ensures a broad and comprehensive capture of HCM cases; however, this methodology is inherently limited by the accuracy of clinical coding and potential variability between healthcare facilities. Ethical considerations were prioritized, and the study adhered to data protection regulations, ensuring patient anonymity throughout the analysis.

The registered incidence of HCM was defined as the number of newly diagnosed patients each year who appeared for the first time in the NFZ database with the applicable ICD-10 codes (I42.1 or I42.2). The registered prevalence was calculated as the total number of patients who appeared at least once in the NFZ database with these ICD-10 codes and were alive as of December 31 of the respective year. Mortality data were obtained from the Polish public healthcare system and the Ministry of Digitization for the years 2016–2021, ensuring a comprehensive assessment of outcomes across the study period.

Epidemiological indicators, including incidence, prevalence, and mortality, were standardized by age, gender, and place of residence, using population data from the Central Statistical Office to provide nationally representative metrics. Survival analysis was conducted to evaluate patient outcomes, with survival curves generated using the Kaplan-Meier method. Differences in survival curves for the analyzed variables, such as age groups and comorbid conditions, were assessed using the Mantel-Haenszel log-rank test to determine statistical significance. This analytical approach enabled a detailed understanding of the temporal trends in HCM-related epidemiology and patient outcomes in Poland during the study period.

## Results

### Incidence and Prevalence in Poland

**FIGURE 1:**
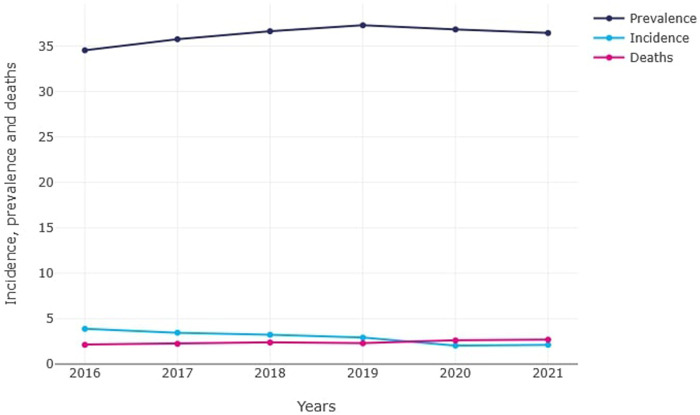
Prevalence, incidence, and mortality due to hypertrophic cardiomyopathy in Poland (Poland, 2016–2021). Prevalence – number of existing hypertrophic cardiomyopathy cases per 100,000 population; Incidence – number of new hypertrophic cardiomyopathy cases per 100,000 population per year; Deaths – number of deaths due to hypertrophic cardiomyopathy per 100,000 population.

In 2016, the registered annual incidence of hypertrophic cardiomyopathy (HCM) in Poland was 1,494 cases (3.89/100,000, 0.004%; men: 55%). In the following years, the number of newly diagnosed cases showed a consistent downward trend: 1,326 cases in 2017 (3.46/100,000, 0.003%; men: 57%), 1,244 cases in 2018 (3.25/100,000, 0.003%; men: 58%), and 1,125 cases in 2019 (2.94/100,000, 0.002%; men: 56%). The most significant decline was observed in 2020, when only 782 new cases (2.04/100,000, 0.002%; men: 58%) were recorded, followed by a slight increase to 808 cases in 2021 (2.11/100,000, 0.002%; men: 59%) [Figure 1].

Despite the decline in new diagnoses, the annual prevalence of HCM exhibited an slightly increasing trend. In 2016, 13,271 cases were registered (34.53/100,000, 0.034%; men: 58%), followed by 13,741 cases in 2017 (35.76/100,000, 0.036%; men: 58%), 14,071 cases in 2018 (36.63/100,000, 0.037%; men: 58%), and a peak of 14,312 cases in 2019 (37.24/100,000, 0.037%; men: 58%). During the pandemic years, prevalence slightly declined to 14,094 cases in 2020 (36.83/100,000, 0.036%; men: 58%) and to 13,880 cases in 2021 (36.23/100,000, 0.036%; men: 59%) [Figure 1].

**FIGURE 2:**
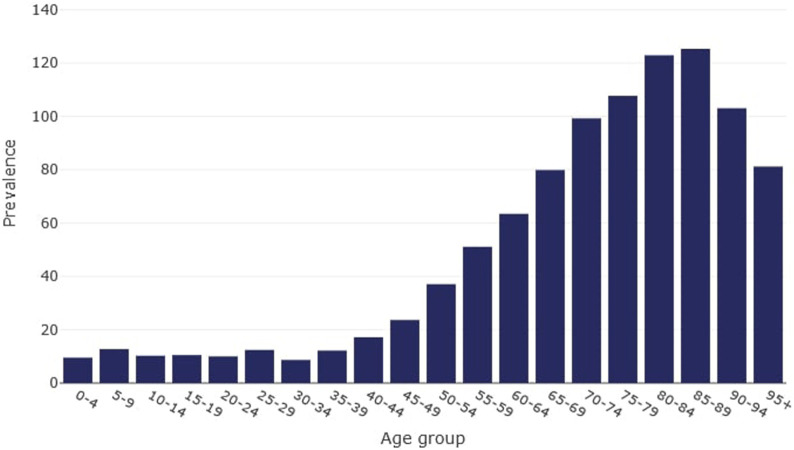
Age-specific prevalence of hypertrophic cardiomyopathy in Poland (Poland, 2016–2021). Prevalence – number of existing hypertrophic cardiomyopathy cases per 100,000 population.

The spectrum of age of the first diagnosis of HCM was presented on Figure 2.

### Mortality

The registered annual mortality related to HCM in Poland increased from 824 deaths (2.14/100,000) in 2016 to 1,022 deaths (2.67/100,000) in 2021. Mortality gradually increased over the years: 856 deaths (2.22/100,000) in 2017, 914 deaths (2.37/100,000) in 2018, 884 deaths (2.29/100,000) in 2019, and 1,000 deaths (2.61/100,000) in 2020. In 2020, the number of deaths exceeded the number of newly diagnosed cases.

However, despite these improvements, men consistently accounted for a higher proportion of deaths compared to women, with mortality rates ranging from 53.4% in 2016 to 56.2% in 2018. During the pandemic years (2020–2021), the gender distribution remained relatively stable (52.9% men vs. 47.2% women in 2020, and 51.6% men vs. 48.4% women in 2021).

### Prognosis

**FIGURE 3:**
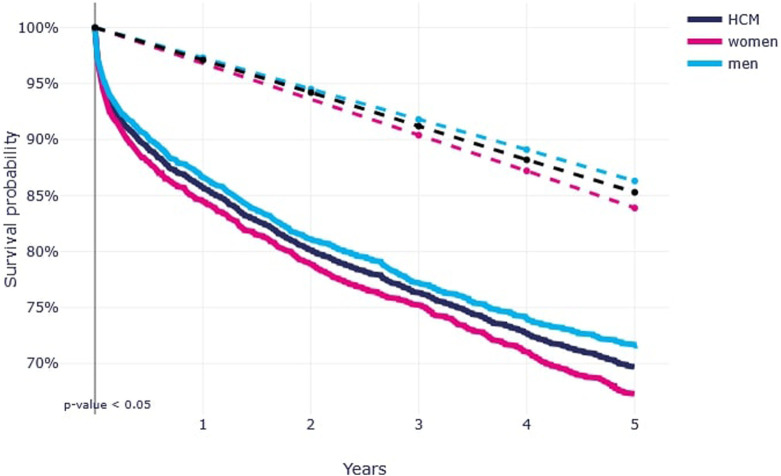
Five-year survival probability among patients with hypertrophic cardiomyopathy, stratified by sex (Poland, 2016–2021).

The 5-year survival probability for HCM was 69%, that was significantly lower than in general population [Figure 3]. Women tend to have slightly lower survival probabilities compared to men.

Survival probability varies significantly by age and sex. The highest 5-year survival rates are observed in younger patients (under 18 years old), while the lowest are seen in the elderly population (75+ years old). Additionally, men tend to have slightly lower survival probabilities compared to women.

### Flows – Paths of HCM Patients

**FIGURE 4:**
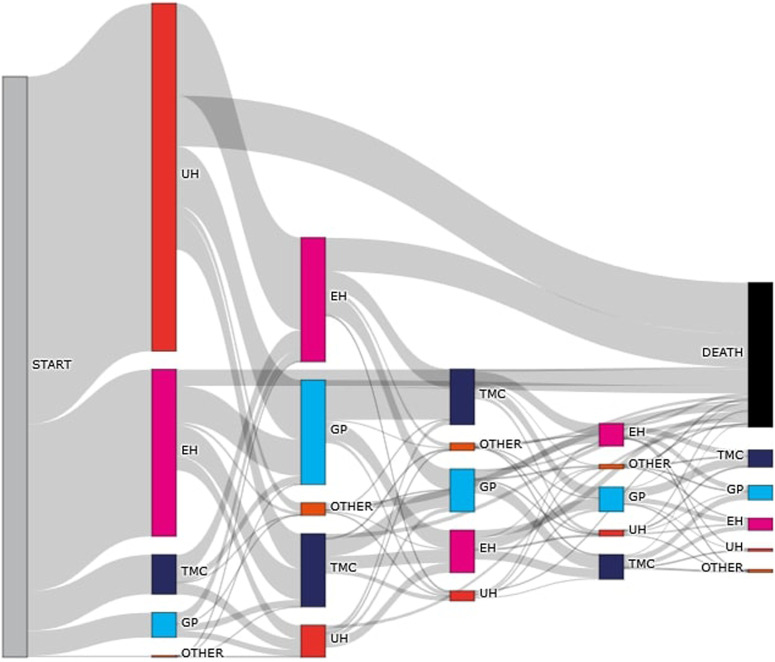
Diagnostic and care pathways of patients with hypertrophic cardiomyopathy in Poland (Poland, 2016–2021). UH: Unplanned hospitalization due to hypertrophic cardiomyopathy exacerbation; EH: Expert hospital (specialized cardiology center); TMC: Outpatient specialist care (ambulatory care); GP: General practitioner; OTHER: Other care providers.

HCM diagnosis as the first registration of HCM code was formulated during hospitalization in 91.4% of patients, in TMC - 6.3%, and in GP - 4.0% of patients; with the diagnosis made during UH in 60.4% and during EH in 31.0% of patients [Figure 4]. Hospital care played a key role in the diagnosis and treatment of HCM, with 93%–96% of cases identified in tertiary care settings. Among 6,773 patients with defined care pathways, over half (52.5%) were treated within a single type of healthcare setting, and 12.8% received care more than once within the same setting. The majority of care episodes involved inpatient hospital care (41.1%), followed by primary healthcare (27.1%) and specialist outpatient services (26.9%). The most common care sequences involved transitions between inpatient, primary, and outpatient specialist care, with inpatient care being the most frequent starting point (33.7%). The average number of care transitions per patient was 5.2, and 4.56 among those whose care pathway ended in death.

### Comorbidities

**FIGURE 5:**
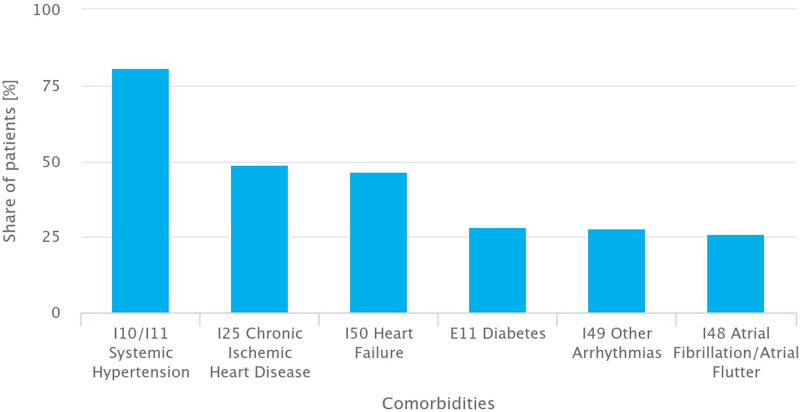
Most common comorbidities among patients with hypertrophic cardiomyopathy in Poland (Poland, 2016–2021).

In patients with HCM in Poland, the most common registered comorbidity was systemic hypertension (ICD-10: I10/I11), diagnosed in 80.9% of cases. Chronic ischemic heart disease (I25) was present in 48.8% of patients, and heart failure (I50) in 46.7%. Other common comorbid conditions included upper respiratory tract infections (J06% – 50.9%), insulin-dependent diabetes mellitus (E11% – 28.2%), cardiac arrhythmias (I49% – 27.7%), and atrial fibrillation or flutter (I48 – ∼25%) [Figure 5].

## Discussion

Hypertrophic cardiomyopathy remains a significant cause of morbidity and mortality, particularly among young people, emphasizing the need for effective diagnostic and therapeutic strategies. The analysis based on data from the National Health Fund reveals great discrepancies between the registered epidemiology of HCM and the estimated number of HCM subjects.

In 2016, the registered annual incidence of hypertrophic cardiomyopathy (HCM) in Poland was 1,494 cases (3.89/100,000, 0.004%) and showed a consistent downward trend. The most significant decline was observed in 2020. The nearly 46% decrease in newly diagnosed cases between 2016 and 2021 can be partially attributed to the COVID-19 pandemic, which disrupted healthcare services, delayed diagnostics, and redirected resources to pandemic-related care.

The observed prevalence of HCM remains significantly lower than international estimates, which suggest that HCM affects 1:250 to 1:500 people (∼75,000–150,000 individuals in Poland). According to our data, the number of registered hypertrophic cardiomyopathy patients was approximately 14,300. This discrepancy highlights the issue of underdiagnosis, which is influenced by limited access to advanced imaging modalities such as cardiac magnetic resonance (CMR) and genetic testing. Additionally, the lack of systematic screening programs for high-risk populations, including individuals with a family history of HCM or unexplained cardiac symptoms, further contributes to the underestimation of the actual number of patients with this condition.

An epidemiological analysis conducted in the United States based on the HealthCore Integrated Research Database (HIRD) from 2013 to 2019 showed that of 16,243,109 patients, 8,526 had a diagnosis of HCM, corresponding to a prevalence of 0.052%. In Poland, the registered prevalence of HCM in 2019 was 37.24 per 100,000 (0.037%), which indicates differences in the frequency of detection and classification of the disease between countries. These differences may result from different coding methods, the availability of diagnostic tools, and variations in healthcare systems [11–14].

Further international comparisons show that the prevalence of HCM in Poland (0.037% in 2019) is consistent with data from the UK (0.035% in 2018) and South Korea (0.031% in 2016) but lower than in Germany (0.07% in 2015) [13, 15–17]. In each of these populations, most cases of HCM were diagnosed in elderly individuals, with a predominance of male patients. These results highlight important differences in HCM recording at the global level, which may be due to differences in access to diagnosis and case reporting.

The registered annual mortality related to HCM in Poland was in the range: 2.14/100,000 in 2016 to 2.67/100,000 in 2021. In 2020, the number of deaths exceeded the number of newly diagnosed cases, highlighting the severe nature of the disease and the potential impact of the COVID-19 pandemic on the care of HCM patients. Between 1990 and 2019, age-standardized mortality decreased in men (from 8/100,000 to 5.6/100,000) and women (from 5.8/100,000 to 3.3/100,000), indicating progress in treatment and disease management over the past decades.

According to previously published Polish and European data, HCM diagnosis in Poland relies predominantly on echocardiography, which is used in approximately 63% of cases, while cardiac magnetic resonance imaging is used in about 33% of patients [18]. However, the lack of systematic use of advanced imaging modalities and genetic testing may limit accurate phenotypic classification and comprehensive risk stratification in routine clinical practice.

In the European RORP registry, the proportion of patients diagnosed with the use of cardiac magnetic resonance imaging differed substantially between countries, reaching approximately 60% in the United Kingdom, while remaining considerably lower in many other regions [19]. Although these data originate from the pre-2020 period, they demonstrate persistent heterogeneity and limited penetration of advanced imaging modalities across Europe, which likely also affects detection rates in Poland.

Hospital care played a key role in the diagnosis of HCM, with 93%–96% of cases identified in tertiary care settings. Patient pathway analysis revealed that a significant proportion of people with HCM experience disease flares and require multiple transitions between outpatient, inpatient, and specialty care, highlighting the complexity of treating this condition and the importance of integrated care strategies. This highlights the need for better coordination between levels of care to improve patient outcomes and reduce the risk of mortality associated with delays or gaps in treatment.

The 5-year survival probability for HCM was less 70, indicating a significantly reduced prognosis compared to the general population. While HCM is often considered one of the more manageable forms of cardiomyopathy, survival in this group remains notably lower. The data confirm that the presence of HCM substantially worsens the prognosis regardless of age and patient group.

Advances in pharmacotherapy, including the widespread use of beta-blockers and calcium channel blockers, as well as septal reduction therapies such as myectomy and alcohol septal ablation, have contributed to improved patient outcomes. Furthermore, the growing use of implantable cardioverter defibrillators for the primary and secondary prevention of sudden cardiac death has significantly enhanced long-term survival. Although the 5-year survival probability for HCM remains lower compared to the general population, the observed decline in age-standardized mortality over recent decades underscores the positive impact of medical innovations.

The data also highlight substantial regional and demographic variability in the incidence, morbidity, and mortality of HCM in Poland. While some regions report higher detection rates, suggesting greater awareness and access to specialized diagnostic services, others may face limitations in healthcare resources, influencing disparities in disease recognition and management. Addressing these differences through the expansion of specialized cardiology centers and improving access to advanced imaging techniques, such as cardiac MRI and genetic testing, could further enhance early detection and personalized treatment strategies.

Additionally, the study results confirm that patients with HCM often present with multiple comorbidities, such as hypertension, heart failure, and atrial fibrillation, which can complicate disease progression and treatment effectiveness. This highlights the necessity for comprehensive, multidisciplinary care strategies that integrate cardiology, electrophysiology, and genetic counseling to optimize patient management. The complex nature of HCM, including its varying phenotypes and genetic underpinnings, further supports the importance of individualized treatment approaches tailored to each patient’s risk profile.

Cardiovascular diseases are of particular importance as they may exacerbate the symptoms of HCM and worsen the prognosis.

A high prevalence of arterial hypertension among patients with hypertrophic cardiomyopathy has also been reported in the European RORP registry, indicating that the burden of hypertension observed in our cohort is comparable with other European populations [9].

The impact of the COVID-19 pandemic on HCM diagnosis and management cannot be overlooked. The temporary decline in newly diagnosed cases during 2020 and 2021 likely reflects disruptions in healthcare services rather than an actual reduction in disease incidence. However, despite these challenges, the resilience of the healthcare system in ensuring continued treatment for existing HCM patients is noteworthy. Moving forward, strengthening healthcare infrastructure to maintain essential cardiology services during future crises will be crucial.

Overall, Poland has demonstrated substantial progress in HCM management, with improved survival rates and better therapeutic interventions. However, ongoing efforts should focus on reducing regional disparities in diagnosis, expanding access to advanced diagnostics, and implementing targeted screening programs for high-risk groups. Future strategies should also prioritize research into novel pharmacological treatments and risk stratification models to further improve patient outcomes [20, 21].

Although increasing availability of cardiac magnetic resonance imaging and genetic testing has been reported in some countries, their routine use in Poland remains limited and unevenly distributed. These diagnostic tools are not systematically implemented in population-based or cascade family screening programs. Consequently, HCM diagnosis in Poland relies mainly on echocardiography in symptomatic patients referred to tertiary centers, leading to persistent underdiagnosis. The declining trend in registered incidence observed even before 2020 likely reflects stable low referral rates, limited awareness in primary care, and the absence of nationwide screening strategies rather than a true reduction in disease occurrence.

Although administrative databases provide valuable information on a large scale, the use of data from the National Health Fund database carries a number of significant limitations. They lack detailed data on individual patient characteristics, such as disease severity, phenotypes or genetic conditions. Additionally, diagnoses of hypertrophic cardiomyopathy may be underestimated or misclassified as heart failure or hypertensive heart disease, leading to underestimation of the true incidence and incidence. Additionally, differences in coding practices between healthcare facilities and regions can introduce inconsistencies in data quality.

### Limitations

The limited availability of advanced diagnostic methods, such as cardiac magnetic resonance imaging and genetic testing, further complicates the accurate diagnosis and differentiation of HCM subtypes. Furthermore, the lack of standardized protocols for the use of CMR limits its diagnostic and prognostic potential in the HCM patient population.

2020 and 2021 should be analyzed carefully given the impact of the COVID-19 pandemic on the healthcare system. The observed decline in the number of HCM diagnoses during this period is likely due to limited access to medical services, delayed diagnosis, and changing priorities in the healthcare system, rather than an actual decline in the incidence of the disease. This highlights the need to build resilient health systems that ensure continuity of essential health services during health crises.

Also noteworthy is the lack of consideration of key clinical outcomes such as quality of life, functional capacity and the risk of arrhythmic complications, which are necessary to fully understand the impact of HCM on patients [19, 20].

The use of administrative data based on ICD-10 coding is associated with a potential risk of misclassification and limited clinical validation. Hypertrophic cardiomyopathy may be underdiagnosed or misclassified as other forms of cardiomyopathy, hypertensive heart disease, or heart failure. In addition, differences in coding practices between healthcare providers may introduce variability in case identification. These factors may lead to an underestimation of the true prevalence and incidence of hypertrophic cardiomyopathy in the analyzed population.

Independent validation of the ICD-10 case definition against individual clinical records (and thus estimation of a study-specific positive predictive value) was not feasible within the National Health Fund administrative database.

Although arterial hypertension was treated as a comorbid condition rather than a diagnostic criterion, hypertensive heart disease may still be misclassified as hypertrophic cardiomyopathy in administrative datasets, which may affect epidemiological estimates.

Arterial hypertension was defined using ICD-10 codes (I10–I11); medication-based proxies could not be applied due to limited access to prescription-level patient data, which may have affected the specificity of hypertension classification.

The National Health Fund administrative dataset does not allow reliable nationwide ascertainment of echocardiography, cardiac magnetic resonance imaging, or genetic testing at the individual patient level; therefore, uptake of these diagnostic modalities could not be quantified in this study.
